# Salmonella Typhi Vaccination Response as a Tool for the Stratification of Risk in Patients with Predominantly Antibody Deficiencies

**DOI:** 10.3390/diagnostics12102423

**Published:** 2022-10-07

**Authors:** Ana Alonso-Larruga, Yvelise Barrios, Andrés Franco, Isabel Suárez-Toste, María José Rodríguez-Salazar, Víctor Matheu

**Affiliations:** 1 Bioquímica Clínica, Laboratorio Central, Hospital Universitario de Canarias, Universidad de La Laguna, 38320 La Laguna, Spain; 2 Inmunología, Laboratorio Central, Hospital Universitario de Canarias, Universidad de La Laguna, 38320 La Laguna, Spain; 3 Neumología, Hospital Universitario de Canarias, 38320 La Laguna, Spain; 4 Hematología, Hospital Universitario de Canarias, 38320 La Laguna, Spain; 5 Alergología, Hospital Universitario de Canarias, Universidad de La Laguna, 38320 La Laguna, Spain

**Keywords:** immunodeficiency, vaccination, polysaccharide, antigen

## Abstract

Predominantly antibody deficiencies are the most frequent type of primary immunodeficiency (PID). Diagnosis requires evaluation of the immune function by distinguishing the presence or absence of a response against polysaccharide antigens. *Salmonella enterica* serovar Typhi-based vaccines have proved to be a suitable tool. We studied a group of patients with suspicion of primary immunodeficiency and classified them by final diagnosis. We analyzed the vaccination response to *S.* Typhi and other immune biomarkers and clinical data. The aim of this study was to classify patients regarding the intensity of their immune response measured as the difference between specific immunoglobulin G levels before and after vaccination and antibody levels in the post-vaccination sample in order to improve clinical decisions regarding follow up and treatment of immunodeficiency patients. We established four groups of response: Non responders (NR), Low responders (LR), Intermediate responders (IR), and High responders (HR), where we found differences in IgG, IgG1, IgG2, IgG4, IgA, IgA1, IgA2, and IgM, and where the finally achieved diagnosis was also different and corresponding to the level of vaccination response.

## 1. Introduction

Clinical manifestations revealing an impaired immune response, such as recurrent or difficult to treat infections, autoimmunity, lymphoproliferation, granulomatous processes, atopy, and malignancies, could indicate an underlying primary immunodeficiency (PID) [1,2]. Primary immunodeficiency diseases (PID) are a heterogeneous group of more than 400 disorders that affect approximately six million people around the world [1,3]. However, it has been estimated that 70–90% are undiagnosed or misdiagnosed [1]. This is due to the fact that even PIDs caused by the same genetic defect present with heterogeneous clinical symptoms varying from severe forms to those with a better prognosis. [2]. An early diagnosis is fundamental in managing patients, improving their quality of life, and it is also important to the health care system, reducing unnecessary costs due to patient’s reiterated admissions [1,2]. Predominantly antibody deficiencies represent the most frequent PID, accounting for at least 55% of all primary immunodeficiencies [4]. They are caused by intrinsic B cell anomalies that result in decreased B cell numbers, low antibody production (immunoglobulin G (IgG), immunoglobulin A (IgA), and immunoglobulin M (IgM)) or both [5]. Diagnosis is based on a characteristic clinical presentation and laboratory tests to assess immune function, which includes testing the response to antigens [2]. T-cell dependent immunity can be evaluated by using protein antigens in either conjugated or protein-based vaccines. On the other hand, T-cell independent immunity can be evaluated by using polysaccharide antigens contained in polysaccharide vaccines [6,7,8]. Pneumococcal polysaccharide vaccines (PPSV) have been the gold standard for this evaluation [7,9] as they are usually well tolerated and are recommended for all children, adults older than 65 years, and high-risk groups. The use of vaccination with pneumococcal conjugate vaccines (PCV) as a routine clinical practice has worked in detriment of the diagnosis of immunodeficiency, due to shared pneumococcal serotypes contained in both vaccines. This has led to increased basal levels of anti-pneumococcal antibodies in individuals where an increase of the immune response after a second exposure may be difficult to interpret [10,11]. Thus, clinicians find themselves in a situation where few tools are available to diagnose antibody deficiencies [12].

As a result, new possibilities have been investigated, such as *Salmonella enterica* serovar Typhi vaccines. The virulence antigen (Vi) is a capsular polysaccharide (ViCPS) in *S.* Typhi that is considered a neoantigen in our population and has been successfully used before in vaccines to control infections [10,13,14]. As with PPSV vaccines, diagnosis requires measuring antibody production before vaccination and four weeks after, and calculating the difference between the measured titers expressed as fold increases (FI). Recent studies have shown that response against *S.* Typhi ViCPS vaccine performed better than the global (non-specific type) responses of 11 pneumococcal antibodies for the discrimination of patients with common variable immunodeficiency (CVID) and hypogammaglobulinemia of healthy control subjects [11,15]. The EMPATHY study established a cut-off value of 3 FI and IgG of 32 U/mL in post-vaccination samples [15]. Another study found that a response ratio of only 2 FI had a sensitivity and specificity of 100% to distinguish control subjects from a group of patients previously diagnosed with antibody deficiency [16]. Besides its role in diagnosis, the same study reported that vaccination with *S.* Typhi could be useful in monitoring IgG therapy (IgGpv), because commercial IgG preparations did not contain measurable anti ViCPS antibodies due to a lack of immunization in our environment [16]. Our group published a study of vaccination in healthy individuals where similar results were obtained [11]. IgG therapy is expensive, a limited resource, and a long-term necessity for PID patients, which has to be used efficiently. Nowadays, it is recommended as a replacement therapy in patients with antibody production deficiencies (a) with IgG < 150 mg/dL, or (b) with hypogammaglobulinemia and poor polysaccharide vaccination response. However there is a group of patients in between with milder presentations where the decision to treat or not, to use intravenous immunoglobulin (IgIV) or antibiotic prophylaxis, is not so clear [17,18]. Several formulas have been proposed to try to objectively make this decision, combining clinical and laboratory data [17].

The aim of this study was to validate *S.* Typhi vaccination in the diagnosis of PID patients and to subclassify PID patients regarding the intensity of immune response against *S.* Typhi vaccination measured in sIgG post-vaccination titers to stratify patients risks in addition to previously published criteria, so we can improve monitorization and indication of IgIV in PID patients depending on their needs.

## 2. Materials and Methods

### 2.1. Study Design

We reviewed all patients older than 15 years old that were submitted to the Primary Immunodeficiency (PID) Unit in the Allergy Department of our hospital [14] between January 2019 and April 2020, with clinical manifestations or laboratory data suggestive of immunodeficiency [19]. Following ESID (European Society for Immunodeficiencies) [20], AAAAI (American Academy of Allergy, Asthma and Immunology) and the ACAAI (American College of Allergy, Asthma and Immunology) guidelines and recommendations [12], the standard clinical practice is routinely carried out in these patients, including an immune function evaluation. Data from the evaluation of vaccination response, other immune biomarkers, and clinical records was gathered retrospectively for each patient and organized in an Excel™ spreadsheet.

This study was approved by the Ethics Committee of our Hospital (Register No. CEIM: 2017_82).

### 2.2. Sample Collection, Vaccination, and Biomarkers

#### 2.2.1. Basal/Pre-Vaccination Samples

Peripheral blood samples were collected by venipuncture in a serum tube (Vacutainer, BD SST) and plasma tube (Vacutainer, BD, EDTA 3K) when necessary. A complete immune function study was performed including:

1. Full blood count and lymphocyte populations (B and T) CoulterDxH and Cytomics FC500 (Beckman Coulter, Brea, CA, USA).
2. Basic biochemistry analysis: Cobas c6000 (Roche Diagnostics, Basel, Switzerland).
3. Immune analysis: Immunoglobulin subtypes (Immunoturbidimetry, Cobas c8000, Roche Diagnostics, Basel, Switzerland) and subclasses (Nephelometry and Turbidimetry, Reference Laboratory), CH50 (Immunoassay, Reference Laboratory), C3 and C4 (Cobas c6000, Roche Diagnostics, Basel, Switzerland).
4. Specific anti-*S.* Typhi Vi IgG antibodies were measured by the VaccZyme™ Human Anti-*Salmonella* Typhi Vi IgG Enzyme Immunoassay Kit (Binding Site Group Ltd., Birmingham, UK) on an AP22 IF, automated system for the ELISA and IFA methods. The VaccZyme™ assay measures the specific IgG against the virulence factor Vi of *S.* Typhi, with a measurement range of 7.4–600 U/mL. For values ≤ 7.4 U/mL, an empirical 7.4 U/mL value was attributed for fold increase calculations. Serum samples were stored at −20 °C after centrifugation (3600 rpm/10 min) and identified with an anonymous barcode.
5. Similarly, the response to vaccination against the pneumococcal conjugate vaccine, 13-valent Prevnar13™ (Pfizer, Berlin, Germany), was measured in pre-vaccination samples and four weeks later in post-vaccination samples, by the VaccZyme™ Anti-PCP IgG Kit EIA (Binding Site Group Ltd., Birmingham, UK), with a measuring range from 3.3 to 270 mg/L.

#### 2.2.2. Vaccination

Vaccination consisted in intramuscular administration by deltoid puncture of a dose of 0.5 mL of sterile solution containing the cell surface Vi polysaccharide extracted from *Salmonella enterica* serovar Typhi (Sanofi Pasteur SA, Lyon, France).

#### 2.2.3. Post-Vaccination Samples

Peripheral blood samples were collected by venipuncture in a serum tube (Vacutainer, BD SST), 4 weeks after vaccination. Specific anti-*S.* Typhi Vi IgG antibodies and specific anti-PCP IgG antibodies were measured as explained in Section 2.2.1. Serum samples were stored at −20 °C after centrifugation (3600 rpm/10 min) and identified with an anonymous barcode.

### 2.3. Clinical Data

Clinical data were collected retrospectively by reviewing patients’ medical records. Search items were clinical or laboratory data indicating the presence or absence of recurrent respiratory infections, severe bacterial infections or sepsis, cutaneous abscesses, chronic or recurrent diarrhea of infectious etiology, severe or recurrent viral infections, mucocutaneous candidiasis, or the presence or absence of other recurrent infections.

### 2.4. Statistical Analysis

Clinical and laboratory data were organized in a Microsoft Excel™ spreadsheet. Samples were divided into four groups regarding intensity of vaccination response as a result of the difference between IgG anti-*S.* Typhi levels post- and pre-vaccination, and measured in fold increases (FI).

Statistical analysis was carried out in Jamovi v.2.2.5 program [21]. First, descriptive analysis for categorical (Table 1a) and continuous (Table 1b) variables was performed using the ClinicoPath tool. Second, a Kruskal–Wallis test was performed to examine the differences between groups for each variable. The statistically significant value was set at *p* = 0.05.

A Dwass–Steel–Critchlow–Fligner pairwise comparison was carried out to examine the differences between the groups. 

## 3. Results

### 3.1. Subjects

Specific anti-*S.* Typhi Vi IgG antibody levels pre- and post-vaccination were obtained in a total of 60 patients, where 36 were women (60%) and 24 were men (40%). The mean age was 48.4 ± 16.3. (Median: 51.5 [Min: 15–Max: 80]). No differences were found between group medians regarding age and sex (*p* = 0.336 and *p* = 0.337).

### 3.2. Groups of Immune Response after Vaccination

Patients were organized in groups based on the intensity of response against *Salmonella* Typhi vaccine, measured in FIs (anti-*S.* Typhi IgG (U/mL) levels in the post-vaccination sample versus the pre-vaccination sample (IgGpost/IgGpre)) and based on the anti-*S.* Typhi IgG (U/mL) levels in the post-vaccination sample.

Using this criteria, four groups were obtained: patients with </=3 FI were considered Non responders [15], and patients with >3 FI were divided into the Low, Intermediate, or High responders groups depending on the anti-*S.* Typhi IgG (U/mL) levels in the post-vaccination samples (Table 2).

Intervals were chosen after previously published studies [9] and our own research of immune response to *S.* Typhi vaccination in healthy individuals [11].

In the present study, pre-vaccination levels of specific IgG anti-*S.* Typhi were obtained in 60 individuals where the median was 7.40 U/mL and the mean was 11.60 U/mL. For each group, the median and mean were: 7.40 and 7.40 U/mL for Non responders (Group 0); 7.40 and 7.81 U/mL for Low responders (Group 1); 7.95 and 8.50 U/mL for Intermediate responders (Group 2); and 11.05 and 17.20 U/mL for High responders (Group 4). (Table 3). Regarding post-vaccination samples, the total median was 97.00 U/mL and the total mean was 205.00 U/mL. For each group, the median and mean were: 7.80 and 8.59 U/mL for Non responders (Group 0); 38.80 and 40.48 U/mL for Low responders (Group 1); 96.95 and 106.49 U/mL for Intermediate responders (Group 2); and 477.05 and 436.05 for High responders (Group 4) (Table 3).

**Figure 1:**
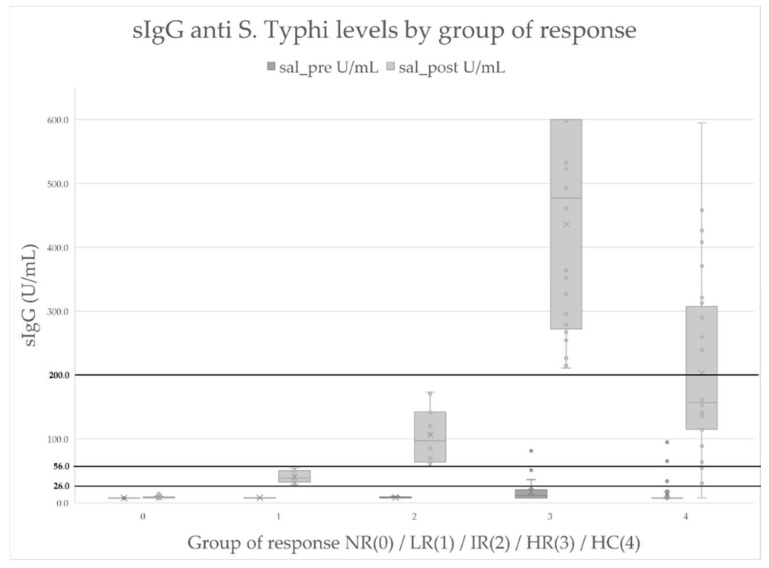
sIgG levels (U/mL) in pre-vaccination (sal_pre) and post-vaccination (sal_post) samples for each group of response and healthy controls. Darker lines show post-vaccination IgG upper limit levels for each group of response. sIgG: specific IgG anti-*S.* Typhi; NR (0): Non responders; LR (1): Low responders; IR (2): Intermediate responders; HR (3): High responders; HC (4): healthy controls.

In a control group of 30 healthy individuals, basal/pre-vaccination levels of specific IgG anti-*S.* Typhi Vi were measured (median: ≤7.4 U/mL; mean: 13.65 U/mL). A total of 27 out of the final 28 of those individuals (96%) showed an increase of anti-*S.* Typhi Vi IgG levels after 4 weeks in post-vaccination samples (median 161.10 U/mL, mean 204.20 U/mL) (Table 3). All of the sIgG levels obtained for each group are represented in Figure 1.

### 3.3. Laboratory and Clinical Data Differences between Groups

The results of laboratory tests and clinical data were organized, separating categorical variables from continuous variables (Table 1). Statistical analysis was carried out (see Section 2.4) to investigate if there were significant differences between the four groups.

**Figure 2:**
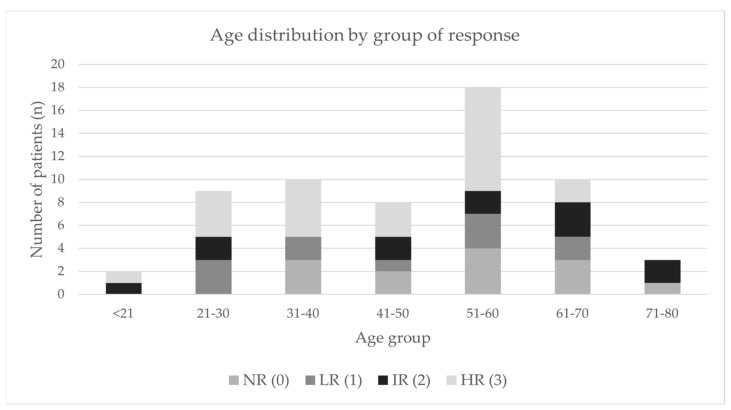
Distribution of patients regarding their age (in years) for each group. NR (0): Non responders; LR (1): Low responders; IR (2): Intermediate responders and HR (3): High responders.

The age distribution of patients for each group is shown in Figure 2.

**Figure 3:**
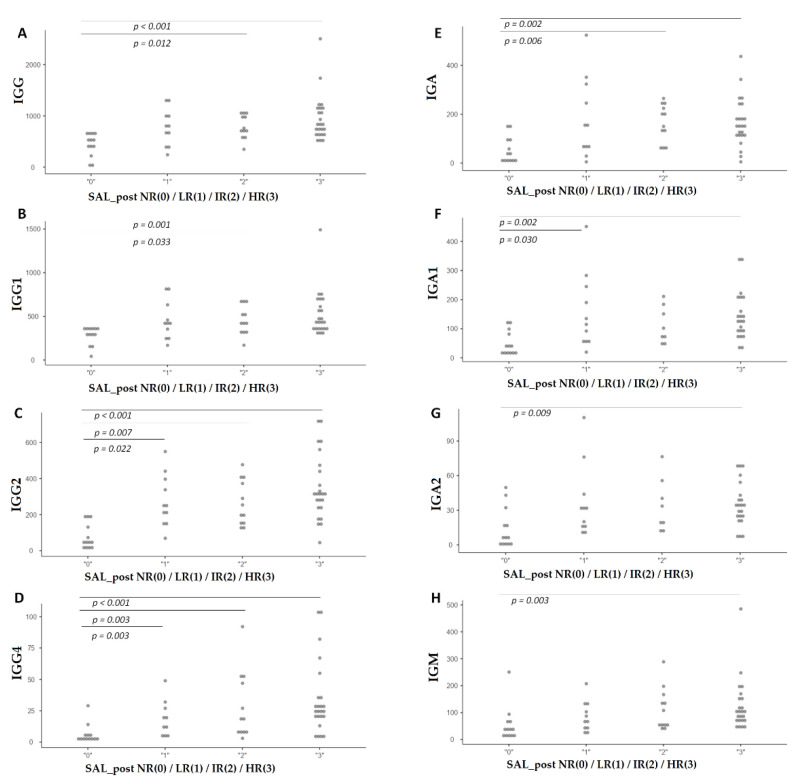
Immunoglobulin levels (mg/dL) for each group of response Sal post NR (0): Non responders; L (1): Low responders; I (2): Intermediate responders; and H (3): High responders. *p* value is shown when statistically significant differences were found between groups. (**A**): IgG; (**B**): IgG1; (**C**): IgG2; (**D**): IgG4; (**E**): IgA; (**F**): IgA1; (**G**): IgA2; and (**H**): IgM.

We found that there were statistically significant differences (*p* < 0.05) between groups in IgG, IgG1, IgG2, IgG4, IgA, IgA1, IgA2, and IgM, especially between the Non responders (Group 0) and High responders (Group 3), and also between the Non responders (Group 0) and Intermediate responders (Group 2). On the other hand, there were also differences between Non responders (Group 0) and Low responders (Group 1), but only in IgG2, IgG4, and IgA1 levels (Figure 3).

On the other hand, no statistically significant differences were found between groups for respiratory tract infections (asthma, otitis, allergic rhinitis, rhinosinusitis, bronchiectasis, bronchitis, and COPD) (*p* = 0.754), number of total pneumonias (*p* = 0.471), number of antibiotics per year (*p* = 0.441), number of hospitalizations per year (*p* = 0.470), bacterial infections (*p* = 0.276), sepsis (*p* = 0.854), abscesses (*p* = 0.676), mucocutaneous candidiasis (*p* = 0.904), diarrhea/abdominal pain (*p* = 0.863), other recurrent infections (*p* = 0.759), joints/bone infections (*p* = 0.802), autoimmunity (rheumatoid arthritis, systemic lupus erythematosus, celiac disease, psoriasis, ankylosing spondylitis, and Raynaud’s) (*p* = 0.559), psychiatric or psychological alterations (*p* = 0.891), malignancies (*p* = 0.805), and any other studied biomarker (*p* > 0.05) in Table 1.

### 3.4. Diagnosis

Patients were later classified by group of diagnosis in common variable immunodeficiency (CVID: *n* = 10, 16.7%), specific antibody deficiency (SAD: *n* = 5, 8.3%), asymptomatic hypogammaglobulinemia (HYPOG: *n* = 32, 53.3%), or patients with no final PID diagnosis (HC: healthy controls; *n* = 13, 21.7%) according to ACAAI definitions [12], as shown in Table 4. 

In the Non responders (Group 0), 10 of 13 patients met the ACAAI criteria of CVID (77%), and 3 of 13 patients met the ACAAI criteria of SAD (23%). In the Low responders (Group 1), 2 of 8 patients met the SAD criteria (18.2%), 8 of 11 met the HYPOG criteria (72.7%), and 1 of 11 was considered HC (9.1%). In the Intermediate responders (Group 2), 9 of 12 patients met the HYPOG criteria (75%) and 3 of 12 were considered HC (25%). In the High responders (Group 3), 15 of 24 patients met the HYPOG criteria (62%) and 9 of 24 were considered HC (38%). There were statistically significant differences regarding the final diagnosis between NR and LR (*p* < 0.001), NR and IR (*p* < 0.001), and NR and HR (*p* < 0.001).

**Figure 4:**
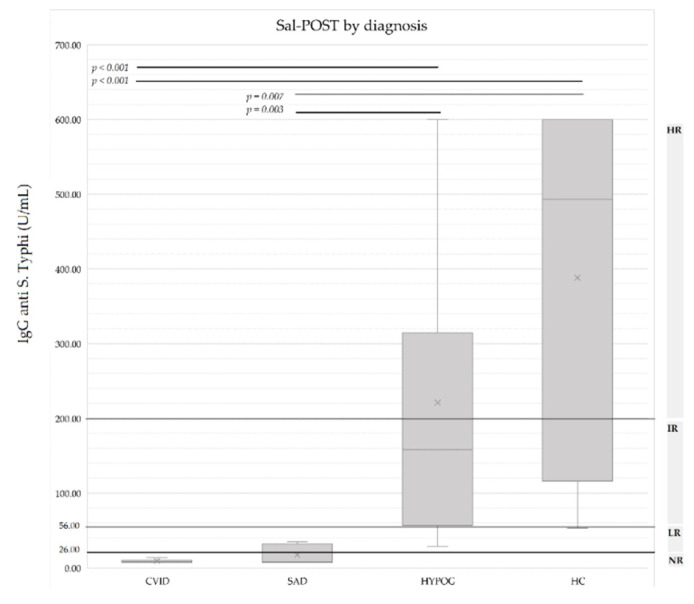
IgG anti-*S*. Typhi levels in post-vaccination samples (U/mL) by group of diagnosis. *p* value is shown when statistically significant differences were found between groups. Darker lines show post-vaccination IgG upper limit levels for each group of response: HR (High responders); IR (Intermediate responders), LR (Low responders); and NR (Non responders). CVID: common variable immunodeficiency; SAD: specific antibody deficiency; HYPOG: hypogammaglobulinemia; HC: healthy controls.

Statistically significant differences were found between IgG anti-*S*. Typhi levels in post-vaccination samples for CVID versus HYPOG (*p* < 0.001), CVID versus HC (*p* < 0.001), and also for SAD versus HYPOG (*p* = 0.003) and SAD versus HC (*p* = 0.007) (Figure 4). The same results were obtained when analyzing the FI (IgGpost/IgGpre).

## 4. Discussion

According to the ACAAI, the laboratory diagnosis of antibody deficiency requires a marked decrease of at least one of the total IgG, IgG1, IgG2, IgG3, IgA, or IgM levels, or failure of IgG antibody response to vaccines [20]. There are four primary immunodeficiencies that largely depend on the analysis of vaccination responses: transient hypogammaglobulinemia of infancy (THI); IgG1, IgG2, or IgG3 subclass deficiency; selective IgA deficiency; and selective antibody deficiency (SAD). Responses to protein or protein-conjugated antigens are typically conserved in these conditions, and response to polysaccharide antigens needs to be performed equally [7].

The diagnosis of SAD is based on antibody levels present after receiving the 23-valent pneumococcal polysaccharide vaccine (PPV23). Even though this test has been the gold standard so far, its interpretation is challenging due to the high basal levels that are frequently found in both healthy populations and PID patients due to routine PCV vaccination recommendations [22]. Patients who already have high baseline antibody concentrations of specific antibodies to a pneumococcal serotype are less likely to have a significant increase in antibody concentrations after immunization. The probability of a fourfold antibody response is drastically reduced as the pre-immunization titer gets higher, depending on the pneumococcal serotype, and the testing of at least six serotypes in the PPV23 is requested [14].

Polysaccharide *Salmonella* Typhi Vi-based vaccination appears as a suitable alternative candidate vaccine to assess the immune response of patients with suspected PIDs [9,10]. Our group has already demonstrated that a sufficient response in fold increases can be achieved in healthy individuals, and preliminarily in PID patients, as well as other groups [9,10,11]. As typhoid fever is not endemic in our environment and vaccine recommendations are only for travelers to countries with a high risk of infection at destination, only very isolated cases present basal antibody levels against *S.* Typhi [11]. Furthermore, *S.* Typhi vaccines are based on an only serotype, in contrast to multiple serotype variants in pneumococcal vaccines, which leads to a simplified assay which is performed quickly and interpreted easily.

In this study, we also aimed to achieve a stratification of risk for patients regarding their intensity of response to vaccination. Regarding SAD, a classification in severe, moderate, mild, and memory form has been proposed based on patient’s age, the number of normal responses to individual polysaccharide pneumococcal serotypes, and the achieved antibody titer [12]. In our case, using *Salmonella* Typhi polysaccharide antigen, we discriminated our patients into Non responders and responders, similarly to previously published studies [15]. However, we aimed to also subclassify patients into Non responders, Low responders, Intermediate responders, and High responders depending on the achieved FI and the anti-*S.* Typhi IgG levels in post-vaccination samples, as shown in Table 2.

The main differences found between the groups turned out to be in IgG, IgG1, IgG2, IgG4, IgA, IgA1, IgA2, and IgM levels. Measuring immunoglobulins is considered a first step in the differential diagnosis of suspected immunodeficiency, and measuring IgG subclasses (IgGSc) is considered a second line step [23]. However, IgGSc concentrations are important when immunoglobulins are within a normal range. IgG1 and IgG3 subclasses are related to immune response against viruses and protein antigens, whilst IgG2 is related to polysaccharide immune response [24].

According to our study, there are differences between some IgGSc in the Non and Low responders, and Intermediate and High responders. This indicates a more severe antibody deficiency, which could lead to a different management or treatment protocol in those groups of patients [24]. We also seem to have differences between groups in the presence of viral infections. However, the number of patients and the retrospective clinical data recollection requires further investigations to confirm these results. 

Patients with SAD might benefit from additional immunization with conjugate vaccines, intensified use of antibiotics, and, in some cases, a period of IgG replacement therapy, which could be also monitored with the *S.* Typhi vaccination [12,18]. Stratification of patients depending on the achieved FI and the anti-*S.* Typhi IgG levels in post-vaccination samples could be an additional help to improve focus and close follow up for those patients in urgent need of medical management.

## Figures and Tables

**Table 1:** Laboratory biomarkers and clinical data collected for each patient. (a) categorical variables; (b) continuous variables. p/a: presence or absence; IgG: immunoglobulin G; IgA: immunoglobulin A; IgM: immunoglobulin M; C3: complement component 3; C4: complement component 4; CH50: total hemolytic complement; LB%: % B cells/ total lymphocytes; LT%: % T cells/ total lymphocytes; LTH%: % CD4 cells/ total T cells; LTS%: % CD8 cells/ total T cells.

**(a) Categorical Variables**
p/a of respiratory tract infections p/a of bacterial infections p/a of sepsis p/a of viral infections p/a of abscesses p/a of mucocutaneous candidiasis p/a of diarrhea/abdominal pain p/a of other recurrent infections p/a of joints/bone infections p/a of autoimmunity p/a of hematological alterations p/a of psychiatric alterations p/a of malignancies
**(b) Continuous Variables**
IgG (mg/dL) IgG1 (mg/dL) IgG2 (mg/dL) IgG3 (mg/dL) IgG4 (mg/dL) IgA (mg/dL) IgA1 (mg/dL) IgA2 (mg/dL) IgM (mg/dL) C3 (mg/dL) C4 (mg/dL) CH50 (UI/mL) Leukocytes LB% and LT% LTH% and LTS% N. of total pneumonias N. of antibiotics/year N. of hospitalizations/year

**Table 2:** Classification of patients in four groups regarding fold increase (FI) calculated as anti-*S.* Typhi IgG levels (U/mL) in post-vaccination (POST) samples divided by IgG levels (U/mL) in pre-vaccination (PRE) samples. *n*: number of patients.

Group	POST vs. PRE IgG Levels	*n*
Group 0: Non responders (NR)	</=3 FI and 0–25 U/mL	13
Group 1: Low responders (LR)	>3 FI and 26–55 U/mL	11
Group 2: Intermediate responders (IR)	>3 FI and 56–200 U/mL	12
Group 3: High responders (HR)	>3 FI and >200 U/mL	24

**Table 3:** Mean values of specific anti-*S.* Typhi IgG levels (U/mL) in healthy individuals (control group) and suspected PID patients of this study in total and divided by groups. *n*: number of patients; PRE: pre-vaccination (basal) samples; POST: post-vaccination samples.

Group	PRE (U/mL)		POST (U/mL)		*n*
	Median	Mean	Median	Mean	
Healthy individuals	7.40	13.65	161.10	204.20	30
Suspected PID patients	7.40	11.60	97.00	205.00	60
Group 0: Non responders	7.40	7.40	7.80	8.59	13
Group 1: Low responders	7.40	7.81	38.80	40.48	11
Group 2: Intermediate responders	7.95	8.50	96.95	106.49	12
Group 3: High responders	11.05	17.20	477.05	436.08	24

**Table 4:** Patient diagnosis. HC: healthy controls; HYPOG: hypogammaglobulinemia; SAD: specific antibody deficiency; CVID: common variable immunodeficiency; *n*: number of patients; %: number of patients in percentage.

Diagnosis	IgG	IgM	IgA	Vaccination Response (T Independent) 3× FI	Vaccination Response (T Dependent) 3× FI
HC	N	N	N	N	N
HYPOG *	↓/N	↓/N	↓/N	N	N
SAD	N	N	N	↓	N
CVID	↓ **	↓	↓	↓	↓

* at least one below the normal range for >15 years old (IgG: 800–1800; IgM: 65–265; IgA: 9–104 mg/dL). ** < 500 mg/dL.

## Data Availability

The data presented in this study are available on request from the corresponding author.
